# When surgery is not an option: case report of transcatheter valve-in-valve replacement for mitral valve dysfunction

**DOI:** 10.1186/s43044-025-00646-y

**Published:** 2025-05-20

**Authors:** Yakup Alsancak, Hasan Kan, Ahmet Seyfettin Gürbüz, Nergiz Aydın, Muhammed Fatih Kaleli

**Affiliations:** 1 Necmettin Erbakan University, Konya, Turkey; 2 Safranbolu State Hospital, Karabuk, Turkey

**Keywords:** Bioprosthetic valve dysfunction, Thoracic radiotherapy, Transcatheter mitral valve replacement, Transseptal approach, Valve-in-valve

## Abstract

**Background:**

Heart valve diseases affect over 100 million people globally, with mitral regurgitation being the most common in developed countries. Bioprosthetic heart valves, frequently used for replacement, typically last 10–15 years before degeneration. Repeat open-heart surgery for valve replacement poses high risks, especially in older or high-risk patients. Following the success of transcatheter aortic valve replacement, transcatheter mitral valve-in-valve replacement has emerged as a less invasive alternative for patients deemed inoperable due to high surgical risks.

**Case presentation:**

We report the case of a 69-year-old male with a history of mitral bioprosthetic valve replacement and thoracic radiotherapy who presented with shortness of breath and NYHA class 3 functional capacity. Echocardiography revealed bioprosthetic valve dysfunction with a mean gradient of 13 mmHg and pulmonary artery pressure of 70 mmHg. Given his high surgical risk (The Society of Thoracic Surgeons score 10.9%, EuroScore2 9.8%) and prior thoracic radiotherapy, a transcatheter valve-in-valve procedure was planned. A 29 mm MyVal valve was successfully implanted via a transseptal approach, resulting in complete resolution of mitral regurgitation and a mean gradient of 3 mmHg post-procedure. The patient was discharged without complications and reported improved functional capacity (NYHA class 1) at follow-up.

**Conclusion:**

This case highlights the successful application of transcatheter valve-in-valve replacement for a patient with bioprosthetic mitral valve dysfunction who was at high surgical risk. The procedure, performed using a transseptal approach with a 29 mm MyVal valve, resulted in significant symptomatic and hemodynamic improvement with no complications. The patient’s functional capacity improved dramatically, and follow-up imaging confirmed the effective functioning of the new valve. This case supports the viability of transcatheter techniques as a preferred alternative for inoperable patients with mitral valve dysfunction, contributing valuable insights to the growing field of minimally invasive cardiac interventions. As technology advances, transcatheter solutions are expected to offer safer and more effective treatments for bioprosthetic valve failures.

**Supplementary Information:**

The online version contains supplementary material available at 10.1186/s43044-025-00646-y.

## Background

Heart valve diseases affect more than 100 million people worldwide, and mitral regurgitation is the most common heart valve disease in developed countries [1]. Treatment options for patients with symptomatic or some asymptomatic advanced-stage valve disease typically include valve replacement or repair [2]. Bioprosthetic heart valves, which can be implanted via both open surgery and transcatheter approaches, have seen an increase in their usage in recent years. However, bioprosthetic valves can degenerate over time, with their durability in the mitral position typically ranging from 10 to 15 years [3]. Although open surgery remains a treatment option for degenerated bioprosthetic valves, the risk of repeat surgery is high due to increased mortality or morbidity in certain patients. Following the success of transcatheter aortic valve replacement (TAVR), transcatheter options have emerged for the treatment of mitral valve pathologies. Transcatheter mitral valve implantation for degenerated bioprosthetic valves has become an alternative treatment, particularly for patients at high risk of undergoing repeat open-heart surgery. The case involved the use of a MyVal valve for mitral valve-in-valve (MVIV) replacement, a device primarily used for TAVI, showcasing its versatility and potential in mitral positions.

## Case presentation

A 69-year-old male patient presented to our clinic with shortness of breath and exercise intolerance. His history included three hospital admissions for pulmonary edema and one for atrial tachycardia in the past six months. His functional capacity was NYHA class 3. The patient had a history of mitral bioprosthetic valve replacement with a 29 mm Sorin Pericarbon (Sorin, Saluggia, Italy) valve due to severe mitral regurgitation secondary to chord rupture 16 years ago. He also had a history of thoracic radiotherapy due to lung cancer ten years ago.

**Fig. 1:**
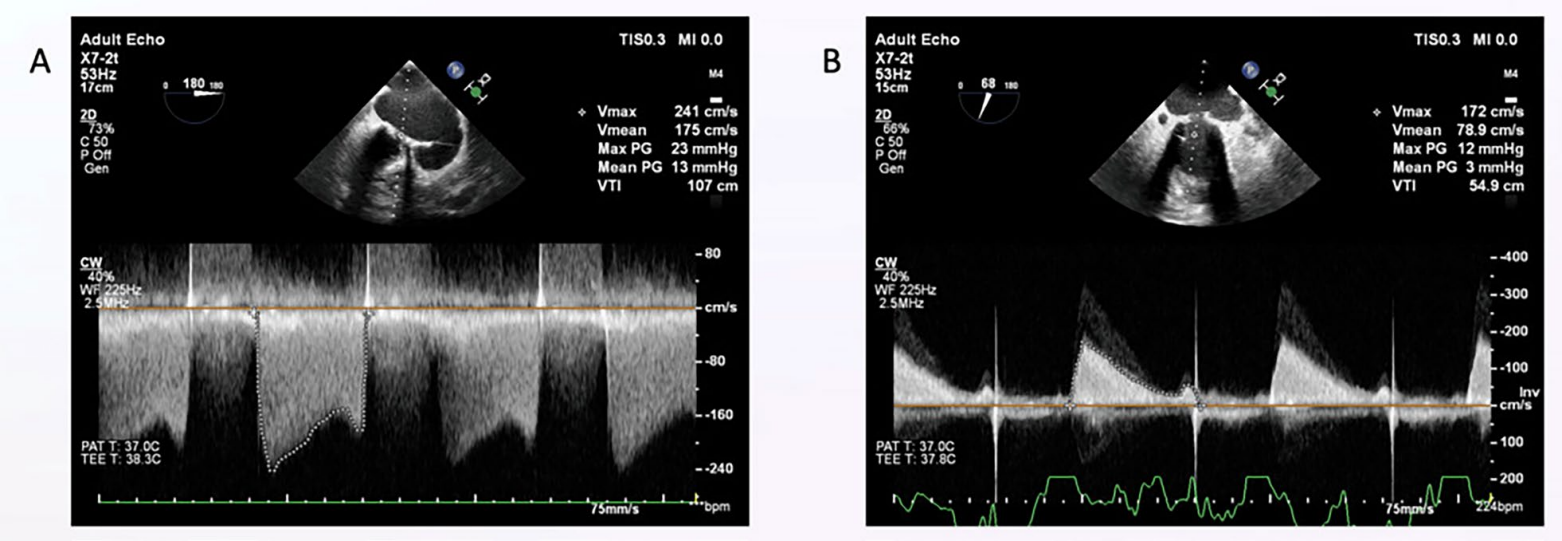
**A**: Bioprosthesis mitral valve gradients before procedure, **B**: Bioprosthesis mitral valve gradients after procedure

The patient was in atrial fibrillation and was receiving medical therapy with warfarin, metoprolol, and furosemide. Echocardiography showed an ejection fraction of 55%, with thickened mitral leaflets and mild mitral regurgitation. The mean mitral valve gradient was measured at 13 mmHg, the valve area was 0.7 cm^2^, and moderate functional tricuspid regurgitation was observed, with the systolic pulmonary artery pressure at 70 mmHg (Video 1*–*2*:* Mitral Valve Before the Procedure, Fig. 1A). Pre-procedural transesophageal echocardiography revealed no evidence of thrombus or vegetation, thereby ruling out valve thrombosis and infective endocarditis. The patient was evaluated as having bioprosthetic valve dysfunction, and no serious stenosis was detected in the coronary angiography. The patient was evaluated together with the heart team, and a decision was made for valve replacement. Due to a high surgical mortality risk (The Society of Thoracic Surgeons score 10.9%, EuroScore2: 9.8%) and his history of thoracic radiotherapy, it was considered that surgical intervention might be unsuccessful, and a transseptal transcatheter procedure was planned.

**Fig. 2:**
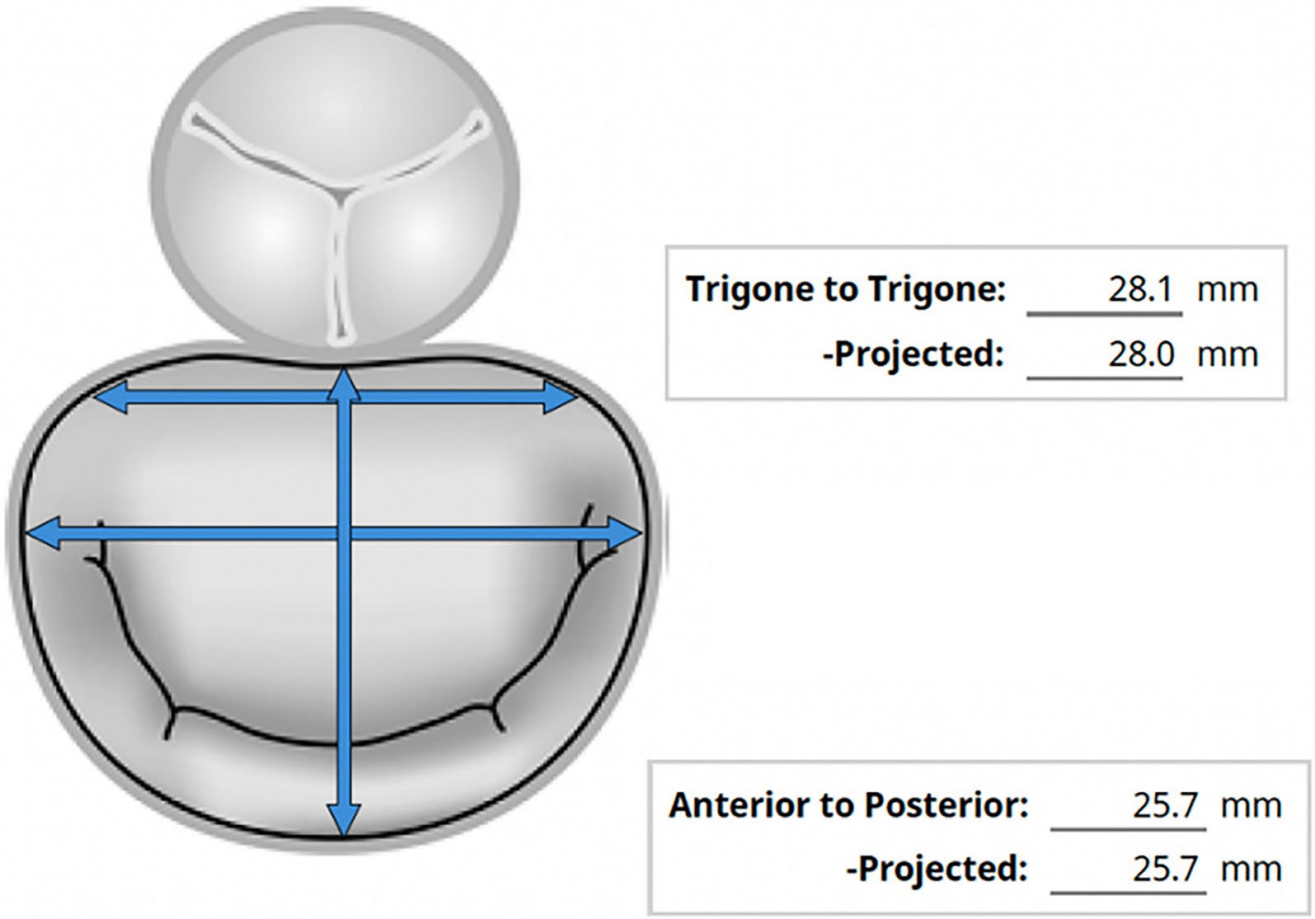
Mitral valve dimensions measured by computed tomography

**Fig. 3:**
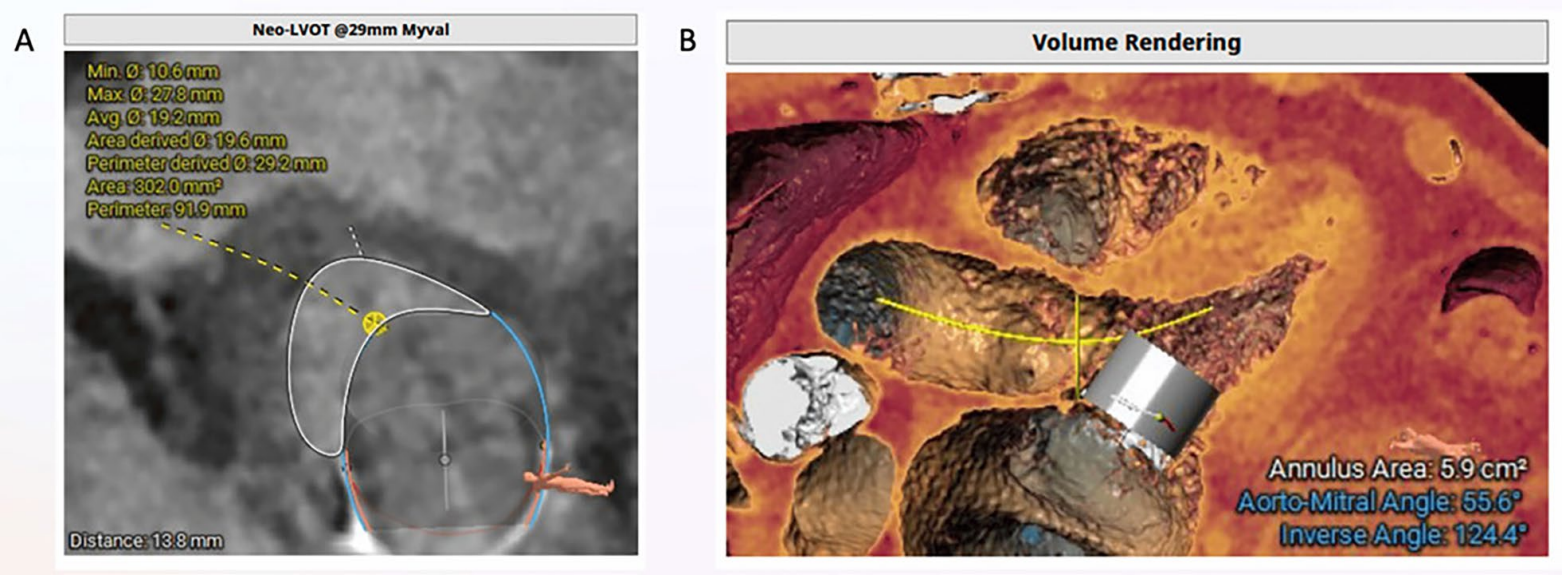
Area of the newly anticipated LVOT (**A**) and the aorto-mitral angle (**B**)

To determine the inner diameter of the existing valve, the size of the new valve to be implanted, and assess the risk of left ventricular outflow tract (LVOT) obstruction, a contrast cardiac computed tomography was performed. The inner diameter of the degenerated bioprosthetic valve was measured at 25 mm, with the mitral valve from trigone to trigone measuring 28 mm and anteroposterior 25.7 mm (Fig. 2). Considering that only short-profile valves can be used in the mitral position, and that higher closing pressures and embolization risks are associated with MVIV [4], it was planned to implant a 29 mm MyVal (Meril Life Sciences Pvt Ltd., Vapi, GJ, IND) valve. The area of the newly created LVOT was calculated to be 302 mm^2^, and the aorto-mitral angle was 55.6 degrees (Fig. 3).

Under general anesthesia and with transesophageal echocardiography guidance, an interatrial septum puncture was performed from the posteroinferior and the region far from the mitral annulus (Video 3: Septum Puncture Guided by Transesophageal Echocardiography). The interatrial septum was dilated with a 12*40 mm peripheral balloon. After puncturing, a guidewire was directed into the left atrium, followed by a catheter. Orientation toward the mitral valve was achieved with the Agilis catheter. A second stiff wire was placed in the ventricle for support. Subsequently, the 29 mm MyVal valve was loaded into the system in the opposite orientation to that of TAVR and advanced into the bioprosthetic valve position (Video 4: Placing the Mitral Valve in the Proper Position). Valve is implanted under rapid ventricular pacing. Post-procedural imaging revealed no mitral regurgitation—either valvular or paravalvular—and no gradient across the left ventricular outflow tract (Video 5–6: Mitral Valve After the Procedure). The overall duration of the procedure was 35 min.

The patient was discharged without complications two days later, with a prescription for rivaroxaban and metoprolol. Post-procedural echocardiography showed a mean mitral valve gradient of 3 mmHg and a reduction in average pulmonary artery pressure to 40 mmHg (Fig. 1B). The patient has been followed for two years, and the most recent echocardiographic evaluation revealed a stable mean gradient of 5 mmHg. Clinically, shortness of breath completely resolved, his functional capacity improved to NYHA class I, and no further hospitalizations were required during the follow-up period.

## Conclusion

The first recommended treatment in the treatment of degenerated bioprosthetic valves is reoperation; however, transcatheter transfemoral valve-in-valve can be recommended in patients with high surgical risk [2, 5]. Although traditional redo mitral valve replacement (MVR) remains the gold standard strategy for degenerative bioprosthetic valves, it is associated with significant complication rates, with mortality rates approaching 11% [6]. Therefore, transcatheter methods should always be considered for patients who may be considered inoperable.

An observational registry study compared surgical options with transcatheter methods for degenerative bioprosthetic valves and found that the transcatheter group had lower mortality and higher technical success rates [7]. Given the continuous increase in life expectancy, it is anticipated that the number of patients requiring intervention due to degenerative bioprosthetic valves will rise in the coming years. Many of these patients may not be surgical candidates due to various factors such as age, frailty, and comorbidities, making transcatheter methods a less invasive treatment option.

Complications that may arise during the procedure should be considered, and rescue strategies should be predetermined. One of the most serious complications of the procedure, LVOT obstruction, should be closely monitored, and in case of its development, emergency surgery, alcohol septal ablation, or the Rescue LAMPOON technique (Laceration of the Anterior Mitral leaflet to Prevent Outflow Obstruction) should be considered [8]. Therefore, the procedure should be performed in comprehensive heart valve centers.

Valves previously used for aortic valve positions can also be utilized for MVIV procedures. Additionally, various valves specifically designed for implantation via a transseptal approach for mitral valves have been developed. The most commonly used valves are the SAPIEN balloon-expandable valves (SAPIEN, SAPIEN XT, and SAPIEN 3, Edwards Lifesciences, Irvine, USA) [9]. Another balloon-expandable valve, the MyVal valve, is primarily used for TAVR, but there are case reports available supporting its use for MVIV [10, 11]. Due to its short profile and wide range of size options, it can be used in carefully selected patients for MVIV.

Although these interventions were initially designed for patients at prohibitive surgical risk, advancements and maturation of these technologies are expected to lead to increased consideration of these techniques and approaches for broader and lower-risk populations.

Successful ViV TMVR can be performed in most cases using a transseptal approach in a minimally invasive manner. This minimally invasive approach has demonstrated promising outcomes, including improved hemodynamics and symptom relief, with a reduced risk of complications compared to traditional redo surgical mitral valve replacement. As the technology and techniques continue to evolve, MVIV is expected to become a more widely adopted treatment strategy, providing a viable alternative for patients who are inoperable or at high risk for conventional surgery. Careful patient selection, pre-procedural planning, and the expertise of comprehensive heart valve centers are crucial to optimizing the success of this procedure and managing potential complications such as left ventricular outflow tract obstruction. The increasing life expectancy and the prevalence of bioprosthetic valve degeneration highlight the growing importance of transcatheter solutions in the future of cardiac care.

## Supplementary Information

Supplementary Material 1. Video 1. Pre-procedural TEE showing stenotic bioprosthetic mitral valve, Transesophageal echocardiography demonstrating thickened, immobile mitral bioprosthetic leaflets with a mean transvalvular gradient of 13 mmHg and mild mitral regurgitation. No thrombus or vegetation observed.

Supplementary Material 2. Video 2. Pre-procedural transthoracic Doppler showing elevated mitral gradient. Transthoracic echocardiography with continuous-wave Doppler confirming elevated mitral valve gradient consistent with bioprosthetic stenosis.

Supplementary Material file 3 Video 3. Septal puncture guided by transesophageal echocardiography. Interatrial septum puncture performed from the posteroinferior region under TEE guidance. A 12×40 mm peripheral balloon was used to dilate the septum.

Supplementary Material 4. Video 4. Valve delivery and positioning across mitral bioprosthesis. The 29 mm MyVal valve, reversed for mitral orientation, is advanced and positioned across the degenerated bioprosthesis with support of stiff ventricular wire.

Supplementary Material 5. Video 5. Post-implantation echocardiography showing no regurgitation. Color Doppler echocardiography after valve deployment showing no valvular or paravalvular mitral regurgitation.

Supplementary Material 6. Video 6. No obstruction in left ventricular outflow tract. Post-procedural echocardiography showing unobstructed LVOT and optimal valve positioning.

## Data Availability

No datasets were generated or analyzed during the current study.

## Abbreviations

ViV: Valve-in-valve
TAVR: Transcatheter aortic valve replacement
LVOT: Left ventricular outflow tract
MVIV: Mitral valve-in-valve
MVR: Mitral valve replacement
LAMPOON: Laceration of the anterior mitral leaflet to prevent outflow obstruction
